# Co-occurrence of Whole-body Muscle Wasting and Respiratory Muscle Weakness Affecting the Clinical Characteristics of Patients with Chronic Obstructive Pulmonary Disease

**DOI:** 10.1298/ptr.E10316

**Published:** 2025-04-23

**Authors:** Atsuyoshi KAWAGOSHI, Masahiro IWAKURA, Yutaka FURUKAWA, Takeshi KERA, Hisashi KAWAI, Shuichi OBUCHI, Keiyu SUGAWARA, Takanobu SHIOYA

**Affiliations:** 1 Department of Rehabilitation, Akita City Hospital, Japan; 2 Department of Environmental Health Science and Public Health, Akita University Graduate School of Medicine, Japan; 3 Department of Physical Therapy, Akita Rehabilitation College, Japan; 4 Department of Physical Therapy, Takasaki University of Health and Welfare, Japan; 5 Research Team for Human Care, Tokyo Metropolitan Institute for Geriatrics and Gerontology, Japan; 6 Department of Physical Therapy, Akita University Graduate School of Health Sciences, Japan

**Keywords:** Muscle strength, Muscular atrophy, Chronic obstructive pulmonary disease, Respiratory muscles

## Abstract

Objectives: The effect of the co-occurrence of whole-body muscle wasting and respiratory muscle weakness on the clinical characteristics of patients with chronic obstructive pulmonary disease (COPD) is unclear. We investigated this association in patients with chronic respiratory diseases. Methods: 47 outpatients with mild to very severe COPD were classified into four groups according to their fat-free mass index and respiratory muscle strength: 19 without muscle wasting and respiratory muscle weakness (normal), 6 with muscle wasting only (MW), 11 with respiratory weakness only (RW), and 11 with muscle wasting and respiratory weakness (MW + RW). We measured their body composition, pulmonary function, lower muscle strength, submaximal exercise capacity, physical activity in daily life, nutritional status, frailty status, and health-related quality of life (QOL). Multiple linear regression analyses examined the association of muscle wasting and/or respiratory muscle weakness with participant characteristics, using each participant’s characteristics as an explained variable adjusted for confounding variables (Normal as the reference). Results: The regression analysis revealed that the percentage of vital capacity, lower muscle strength, submaximal exercise capacity, nutritional status, and frailty status were worse in the MW + RW group than in the Normal group. The MR + RW group had the largest number of variables with significant differences compared to the Normal group. Conclusions: These data suggest that the co-occurrence of whole-body muscle wasting and respiratory muscle weakness was significantly associated with deteriorating clinical characteristics in patients with COPD.

## Introduction

Sarcopenia is registered as a disease code in the 10th edition of the International Classification of Diseases^1,2)^, and it has been reported to be associated with cardiovascular and respiratory diseases^3,4)^ and subsequent progression to a state requiring long-term care and death^5,6)^. In recent years, the concept of respiratory sarcopenia has begun to be newly proposed. Peak expiratory flow rate (PEFR) is associated with a decrease in muscle mass, and respiratory sarcopenia can be a risk factor for requiring long-term care insurance certification^7)^. Inspiratory muscle strength is correlated with lower extremity function and exercise capacity in patients with chronic obstructive pulmonary disease (COPD)^8)^. Nagano et al.^9)^ have reported the concept of respiratory sarcopenia, which is the co-occurrence of whole-body sarcopenia, defined as sarcopenia^1)^, and respiratory muscle weakness. This was recently issued as probable respiratory sarcopenia by the joint meeting of four academic societies^10)^. Probable respiratory sarcopenia can be associated with further worsened clinical characteristics of COPD and adversely affect their prognosis because each factor is an established risk factor for poor prognosis. However, there is insufficient evidence for the presence of probable respiratory sarcopenia and its associations with clinical characteristics in patients with COPD.

Therefore, the purpose of this study is to describe the prevalence of the co-occurrence of whole-body muscle wasting, defined by the fat-free mass index (FFMI), and respiratory muscle weakness, and to explore the associations between these factors and the clinical characteristics of patients with COPD. Revealing this question can be useful information for recognizing the importance of the clinical significance of a probable-respiratory sarcopenia diagnosis and its management.

## Methods

### Participants and study design

This study was a cross-sectional study. 47 outpatients with mild to very severe COPD undergoing home-based pulmonary rehabilitation with low-intensity exercise at Akita City Hospital were enrolled in the study. Medication prescriptions for the patients included long-acting muscarinic antagonists or/and β_2_ agonists, inhaled corticosteroids, and short-acting β_2_ agonists as necessary. All patients were retired and met the following inclusion criteria: (1) diagnosis of COPD according to the Global Initiative for Chronic Obstructive Lung Disease^11)^; (2) being in a stable condition with no infection or exacerbation of COPD in the prior 3 months; (3) having no severe and/or unstable cardiac disease, orthopedic disease, or mental disorder that could impair their activities in daily life. The detailed comorbidities in the patients are as follows: 4 osteoporosis, 5 diabetes, 10 chronic heart failure, 14 hypertension, 3 anemia, 8 hyperlipidemia, 1 chronic renal failure, 5 arthritis, and 7 spinal canal stenosis.

### Study protocol

The assessment of body composition, pulmonary function, skeletal muscle strength (quadriceps femoris muscle force [QF]), inspiratory muscle strength (maximal inspiratory pressure [MIP]), submaximal exercise capacity (six-minute walk distance [6MWD]), physical activity in daily life (steps), dyspnea (modified Medical Research Council [mMRC]^12)^), disease-specific health-related quality of life (COPD Assessment Test [CAT]^13)^), nutritional status (Mini Nutritional Assessment-short form [MNA-SF]^14)^), and frailty status (Kihon checklist [KCL]^15)^, Cardiovascular Health Study [the J-CHS]^16)^) were performed by well-trained physical therapists who were independent of this study. The methods for these outcomes are described in detail in Appendix 1.

Whole-body muscle wasting was assessed using FFMI; FFMI <16 kg/m^2^ for males and <15 kg/m^2^ for females^17)^, and respiratory muscle weakness was evaluated using MIP; <60 cmH_2_O for males and <40 cmH_2_O for females^18)^. Participants were classified into four groups: no muscle wasting and no respiratory weakness (Normal), muscle wasting and no respiratory weakness (muscle wasting [MW]), no muscle wasting and respiratory weakness (respiratory weakness [RW]), and muscle wasting and respiratory weakness (MW + RW).

This study was approved by the medical ethical committee of Akita City Hospital (Date: March 28, 2019, Approval No. 109). Written consent was obtained after the objective and content of the study were orally explained to the participants. All participants were informed that their privacy would be sufficiently protected. All procedures were performed in accordance with the research ethics guidelines of the Declaration of Helsinki^19)^.

### Statistical analysis

Statistical analyses were performed using the R statistical software version 4.0.5 (R Project for Statistical Computing) within RStudio version 1.4.1106 (RStudio, Boston, Massachusetts, USA). We chose complete case analysis to deal with missing data. Normality in data distribution was assessed using the Kolmogorov–Smirnov test with p-values <0.05 considered significant.

Multiple linear regression analyses were performed to test the association of muscle wasting and respiratory muscle weakness status with the participant’s characteristics. This analysis can compare each variable of the Normal group with the other three groups respectively. We used each participant’s characteristic as an explained variable, muscle wasting and low respiratory muscle strength status as an explanatory variable (Normal, MW only, RW only, and MW + RW), and age, sex, and disease severity, presented by the percent predicted value of forced expiratory volume in one second (FEV_1_), as biological and pathological confounding variables influencing their prognosis^20)^. We set the Normal group as the reference in the analyses. The participant’s characteristics included the percent predicted value of vital capacity (VC) and forced vital capacity (FVC), mMRC, CAT, 6MWD, QF, steps, MNA-SF, KCL, and J-CHS score. All data analyses should be interpreted as explanatory data analyses because numerous tests were performed.

## Results

From the 47 patients considered in the analysis, 19 (40.4%) were classified as Normal, 6 (12.8%) were classified as MW, 11 (23.4%) were classified as RW, and 11 (23.4%) were classified as MW + RW. The demographic and clinical characteristics of the patients are presented in Appendix 2.

Table 1 presents the association of muscle wasting and respiratory muscle weakness status with the participant’s characteristics after adjustments for age, sex, and lung function. MW and MW + RW groups presented, in comparison with Normal as a reference, significantly higher CAT scores and lower MNA-SF scores. The RW and MW + RW groups presented significantly lower VC%pred and 6MWD. All groups presented lower QF compared to the Normal group. In addition, the MW + RW group only presented lower FVC%pred, higher KCL, and J-CHS scores. The RW group only presented a higher mMRC. The MR + RW group had the largest number of variables with significant differences compared to the normal group.

**Table 1.** Multiple regression analysis for muscle wasting and low respiratory muscle strength status using clinical characteristics

Variables	MW (n = 6)	RW (n = 11)	MW + RM (n = 11)
VC, %pred			
β [95% CI]	−14 [−29, 2.1]	−17 [−30, −3.0]	−26 [−39, −13]
p-value	0.1	0.021	<0.001
FVC, %pred			
β [95% CI]	−12 [−27, 3.4]	−13 [−27, 0.23]	−28 [−41, −16]
p-value	0.14	0.061	<0.001
mMRC			
β [95% CI]	−0.08 [−0.84, 0.68]	0.85 [0.19, 1.5]	0.31 [−0.32, 0.93]
p-value	0.8	0.015	0.3
CAT, points			
β [95% CI]	8.4 [1.6, 15]	1.8 [−4.1, 7.6]	7.1 [1.6, 13]
p-value	0.02	0.6	0.016
6MWD, m			
β [95% CI]	−103 [−205, −1.0]	−139 [−227, −51]	−164 [−248, −80]
p-value	0.055	0.004	<0.001
QF, kg			
β [95% CI]	−16 [−27, −4.5]	−9.9 [−20, −0.27]	−15 [−24, −5.8]
p-value	0.009	0.051	0.003
Steps, steps/day			
β [95% CI]	1014 [−2,271, 4,299]	−1200 [−3,965, 1,564]	−2066 [−4,614, 483]
p-value	0.5	0.4	0.12
MNA-SF, points			
β [95% CI]	−3.1 [−4.8, −1.3]	−0.78 [−2.3, 0.71]	−4.2 [−5.7, −2.7]
p-value	0.001	0.3	<0.001
KCL, points			
β [95% CI]	0.16 [−0.18, 0.51]	0.29 [−0.01, 0.58]	0.36 [0.08, 0.64]
p-value	0.4	0.065	0.017
J-CHS score, points			
β [95% CI]	0.52 [−0.43, 1.5]	0.32 [−0.50, 1.1]	0.87 [0.09, 1.7]
p-value	0.3	0.4	0.035

Regression coefficient (β) adjusted for age, sex, and FEV_1_%pred is expressed as differences from Normal (n = 19) as reference.

CI, confidence interval; FEV_1_, forced expiratory volume in the first second; MW, muscle wasting; RW, respiratory weakness; VC, vital capacity; FVC, forced vital capacity; mMRC, modified Medical Research Council; 6MWD, six-minute walk distance; CAT, COPD Assessment Test; QF, quadriceps femoris muscle force; MNA-SF, Mini Nutritional Assessment-short form; KCL, Kihon Checklist; J-CHS, Japanese version of Cardiovascular Health Study criteria

## Discussion

The results of this investigation revealed a cross-sectional association with clinical features after stratification into whole-body muscle wasting and respiratory muscle weakness status, compared to patients without these conditions, in patients with COPD. Patients with MW + RW presented various deteriorations in physical functions, especially in lung capacity, lower limb muscle strength, and exercise capacity, frailty status, and health-related QOL, which must be recognized as clinical importance of probable-respiratory sarcopenia diagnosis and its management.

VC, mMRC, and 6MWD in the RW group declined compared to the Normal group. The relationship between respiratory function and inspiratory muscle strength has been shown in accordance with previous studies^7)^. Singer et al.^8)^ have reported that reduced respiratory muscle strength was associated with decreased exercise capacity in patients with COPD. The RW group was matched with respiratory muscle weakness due to respiratory dysfunction, as defined in the position paper^10)^. Diaphragmatic dysfunction, which is caused by specific pathologies in patients with COPD (such as lung hyperinflation, oxidative stress, systemic inflammation, and malnutrition), can lead to increased dyspnea by increasing respiratory muscle fatigue and decreasing endurance^21)^.

The MW group presented significantly worse CAT, QF, and MNA-SF scores compared to the Normal group. It has been reported that 25%–40% of patients with COPD are malnourished^22,23)^. The MNA-SF was clearly associated with the malnutrition status of patients with MW, which was presented as low FFMI in this study. Patients with sarcopenia in COPD exhibit reduced respiratory function, exercise tolerance, and quality of life^24)^. The findings in the MW group are in accordance with previous studies. Moreover, the addition of respiratory muscle weakness to MW (MW + RW group) resulted in significantly worse VC%pred, FVC%pred, CAT, 6MWD, QF, MNA-SF, KCL, and J-CHS scores compared to the Normal group, which suggests that the MW + RW group exhibits more indicators of reduced function than the other two groups. This has been shown to deteriorate the clinical features unique to COPD, which have not been shown in previous studies involving healthy older individuals^7,10)^. Since the amount of physical activity tends to decrease, it is thought that there was no significant change in mMRC because the participants were living without dyspnea. This may also have an effect on the significant difference in the index of long-term care risk, which has only been observed in the MW + RW group, who are almost matched as probable respiratory sarcopenia, as suggested by the position paper. Kera et al. have reported that PEFR can be significantly correlated with long-term care insurance certification in community-dwelling older people^7)^. This association, as a link between decreased cough peak flow and aspiration-related pneumonia, has been mentioned. The review reported that the diaphragm is the most important inspiratory muscle and the maximum inspiratory pressure is an independent determinant of survival in patients with severe COPD^21)^. Thus, it may be suggested that conventional sarcopenia, which affects long-term care and mortality, combined with respiratory muscle weakness as expressed by inspiratory muscle strength, is more likely to lead to frailty and the risk of long-term care. As proposed by the position paper^10)^, the reduction of respiratory muscle mass is essential for the definitive diagnosis of respiratory sarcopenia; however, it is difficult to measure in clinical settings. Therefore, probable-respiratory sarcopenia, which can be diagnosed without measuring respiratory muscle mass, leads to greater functional decline than conventional sarcopenia alone and should be a focus of clinical attention.

The prevalence of MW + RW, which is more deteriorating than their individual parameters, was 23.4% in this study. Morisawa et al.^25)^ reported that the prevalence of cases with inspiratory muscle weakness and conventional sarcopenia, similar to those in this study, was observed in 12% of community-dwelling older people. The prevalence in patients with COPD might be higher than that in community-dwelling older people, considering that the prevalence of whole-body sarcopenia is 21.6%^26)^ and their %MIP decreases as the disease progresses^8)^. In addition, it has been reported that there is a case in which respiratory muscle weakness occurs even without whole-body sarcopenia in community-dwelling older people^25)^, which was observed in 23.4% of patients with COPD in the present study. This seems to be the result of some individuals, even if the whole-body muscle mass is maintained, who have diaphragmatic dysfunction due to the pathophysiology peculiar to COPD^21)^.

An important limitation of the present study must be addressed. First, the number of patients recruited in each group was a small sample size in the single-centered study. Therefore, the results do not reflect those of all stable COPD patients. However, the number of samples in the linear regression analyses of this study could satisfy the minimum number of subjects per variable for estimating regression coefficients^27)^. Although age, gender, and disease severity were used as confounding variables in regression analyses, other factors, such as differences in comorbidities, smoking status, and pharmacotherapy, which could have an influence on the association of muscle wasting and respiratory muscle weakness status with the participant’s characteristics, were not taken into consideration to ensure the accuracy of the calculated estimating regression coefficients. In addition, the generalizability of the study results may be poor due to fewer patients with severe COPD especially. Second, the grouping criteria for muscle wasting are based on FFMI rather than skeletal muscle mass index. It is desirable to divide the groups using the criteria of the Asian Working Group for Sarcopenia 2019^28)^ as the position paper suggested. Further studies with a larger sample size are required to conduct multivariate analyses that consider other confounding factors so that the associations with more parameters on the status of respiratory sarcopenia can be clarified.

## Conclusions

In summary, our present findings demonstrated a cross-sectional association between clinical features and the prevalence of the status with whole-body muscle wasting and respiratory muscle weakness in patients with COPD. The MW + RW group, which is almost matched as probable respiratory sarcopenia, as suggested by the position paper, is 23.4% in this study. They had a larger number of variables, including frailty, with significant deterioration compared to the Normal group than the MW and RW groups. Further studies are needed to investigate the association between clinical features and patients with respiratory sarcopenia, stratified using verified criteria, with a larger sample size.

## Acknowledgments

The authors acknowledge the assistance provided by members of the Department of Rehabilitation at Akita City Hospital for data collection. We would also like to thank Editage (www.editage.jp) for editing the English language.

## Funding

Not applicable.

## Conflicts of Interest

The authors declare no conflicts of interest.

## Supplementary Materials

Appendix 1. Outcome measures.

Appendix 2. Demographic and clinical characteristics of the participants with COPD stratified into muscle wasting and low respiratory muscle strength status.
