# Supplementary material for: Co-occurrence of Whole-body Muscle Wasting and Respiratory Muscle Weakness Affecting the Clinical Characteristics of Patients with Chronic Obstructive Pulmonary Disease
Source: Phys Ther Res. 2025 Apr 23;28(2):145–9. doi: 10.1298/ptr.E10316 (PMC12445364; doi:10.1298/ptr.E10316)
Supplement: Appendix 1. — Outcome measures. [file ptr-28-145-s01.pdf]

Appendix 1

Outcome measures

Body height was determined to the nearest 0.1 cm (HP-I, Fukui Doryoki Co; Kyoto, Japan) with subjects standing barefoot. Body weight was assessed with a beam scale to the nearest 0.1kg (Omron Co; Kyoto, Japan), with subjects standing barefoot and in light clothing. Fat-free mass (FFM) was estimated with single-frequency (50 kHz) bioelectrical impedance analysis (DC-320, Tanita, Co., Ltd. Tokyo, Japan). Resistance was measured with subjects in the supine position.

Pulmonary function was assessed by vital capacity (VC), forced vital capacity (FVC), forced expiratory volume in one second (FEV_1), and FEV_1/FVC using a spirometer (FUDAC-77; Fukuda Denshi Co., Ltd., Tokyo, Japan).

Inspiratory muscle strength (maximal inspiratory pressure (MIP) was measured as inspiratory muscle strength using a respiratory dynamometer (Autospiro AS-507, MINATO Medical Science, Co., Ltd., Osaka, Japan) following the American Thoracic Society (ATS)/European Respiratory Society recommendations [1)].

Quadriceps femoris muscle force (QF) was measured as skeletal muscle strength. For QF, the maximum isometric extension and contraction were measured at 0°/sec 80° flexion using Hydro Musculator GT-160 (OG Giken Co., Okayama, Japan) [2)]. For the measurements of exercise capacity, a 6-minute walk test (6MWT) was performed according to the ATS guidelines [3)]. The participants were not encouraged during the 6MWT.

Physical activity (PA) in daily life was assessed using a uni-axial accelerometer, Lifecorder GS (Suzuken Co., Ltd., Aichi, Japan) [4)], which is a small and lightweight activity monitor (625 × 465 × 260 mm, 40 g). The pedometer contains a uniaxial accelerometer to measure the wearer’s energy expenditure and the number of steps every 4 s throughout each day.

The validity and reliability of this device have been proven [4,5]. The participants were instructed to wear the device on their waist belts for 12 hours after waking up every day for 2 to 4 weeks. PA data were then collected and analysed using dedicated software. We excluded participants from the analysis if the number of valid days of activity measurement was less than four or/and if they wore the device for 10 hours or less per day. Since PA decreases during winter, the measurement was performed during seasons other than winter [6].

Dyspnea was assessed using the modified Medical Research Council (mMRC) dyspnea scale [7]. Disease-specific health-related quality of life (QoL) was measured using the COPD Assessment Test (CAT) [8]. Nutritional status was estimated using the Mini Nutritional Assessment-short form (MNA-SF) [9]. MNA-SF includes the following six domains: appetite loss (0–2 points), weight loss (0–3 points), mobility (0–2 points), stress/acute disease (0 or 2 points), neuropsychological impairment (0–2 points), and BMI (0–3 points). According to the total score of MNA-SF, patients were classified into three categories: malnourished (0–7 points), at risk of malnutrition (8–11 points), or a normal nutritional status (12–14 points).

Frailty status was assessed by the Kihon checklist (KCL) [10] and physical frailty was measured on the basis of the Japanese version of the Cardiovascular Health Study (the J-CHS) criteria consisting of five physical components (walking speed, handgrip strength, shrinking, exhaustion, and physical inactivity) as J-CHS score [11]. The KCL is a questionnaire consisting of five subdomains with five items each [10]. Each response indicative of the potential need for preventive care receives a point; this might be a “Yes” to a “negative” question or a “No” to a “positive” question. In the present study, participants with five or more points were considered frail [12].

1) ATS/ERS: ATS/ERS statement on respiratory muscle testing. Am J Respir Crit Care Med. 2002; 166: 518-624.
2) Decramer M, Lacquet LM, et al.: Corticosteroids contribute to muscle weakness in chronic airflow obstruction. Am J Respir Crit Care Med. 1994; 150: 11-16.
3) ATS committee on proficiency standards for clinical pulmonary function laboratories: ATS statements: Guidelines for six-minute walk test. Am J Respir Crit Care Med. 2002; 166: 111-117.
4) Kumahara H, Schutz Y, et al.: The use of uniaxial accelerometry for the assessment of physical-activity-related energy expenditure: a validation study against whole-body indirect calorimetry. Br J Nutr. 2004; 91: 235-243.
5) Schneider PL, Crouter SE, et al.: Accuracy and reliability of 10 pedometers for measuring steps over a 400-m walk. Med Sci Sports Exerc. 2003; 35: 1779-1784.
6) Sewell L, Singh SJ, et al.: Seasonal variations affect physical activity and pulmonary rehabilitation outcomes. J Cardiopulm Rehabil Prev. 2010; 30: 329-333.
7) ATS/ERS: Definition, diagnosis and staging. In: Standards for the diagnosis and management of patients with COPD. American Thoracic Society and European Respiratory Society. 2004; 8-13.
8) Jones PW, Harding G, et al.: Development and first validation of the COPD Assessment Test. Eur Respir J. 2009; 34: 648-654.
9) Van Nes MC, Herrmann FR, et al.: Does the mini nutritional assessment predict hospitalization outcomes in older people? Age Ageing. 2001; 30: 221-226.
10) Japan Ministry of Health Labor and welfare: Manual of services of care prevention benefits. 2014. [cited 08 Nov 2019]. Available from: http://www.mhlw.go.jp/topics/2009/05/tp0501-1.html

11) Sakata S, Arai H.: The revised Japanese version of the Cardiovascular Health Study criteria (revised J-CHS criteria). Geriatr Gerontol Int. 2020; 20: 992-993.
12) Ogawa K, Fujiwara Y, et al.: The validity of the"Kihon Check-list" as an index of frailty and its biomarkers and inflammatory markers in elderly people. Nippon Ronen Igakkai Zasshi. 2011; 48: 545-552.
