# Supplementary material for: Co-occurrence of Whole-body Muscle Wasting and Respiratory Muscle Weakness Affecting the Clinical Characteristics of Patients with Chronic Obstructive Pulmonary Disease
Source: Phys Ther Res. 2025 Apr 23;28(2):145–9. doi: 10.1298/ptr.E10316 (PMC12445364; doi:10.1298/ptr.E10316)
Supplement: Appendix 2. — Demographic and clinical characteristics of the participants with COPD stratified into muscle wasting and low respiratory muscle strength status. [file ptr-28-145-s02.pdf]

Appendix 2. Demographic and clinical characteristics of the participants with COPD stratified into muscle wasting and low respiratory muscle strength status

Variables	Overall (n=47)	Normal (n=19)	MW (n=6)	RW (n=11)	MW+RW (n=11)
Age, yrs	76 (5)	74 (4)	74 (4)	79 (5)	74 (6)
Sex (M/F), n	41/6	19/0	4/2	10/1	8/3
BMI, kg/m^2	21.8 (3.8)	24.2 (2.8)	18.8 (1.7)	23.7 (2.5)	17.5 (1.8)
FFMI, kg/m^2	17.0 (2.7)	18.64 (1.9)	14.7 (0.9)	18.4 (2.3)	14.0 (1.1)
VC, %predicted	89.4 (27.1)	105.8 (20.4)	82.6 (20.9)	85.2 (26.3)	69.0 (26.4)
FVC, %predicted	91.4 (27.9)	106.6 (21.9)	86.4 (17.8)	90.7 (27.9)	68.7 (27.7)
FEV_1/FVC, %	54.2 (18.9)	50.8 (14.3)	55.4 (20.7)	47.0 (14.67)	66.8 (24.5)
FEV_1, %predicted	61.2 (26.7)	68.2 (24.2)	60.4 (27.6)	54.4 (28.7)	56.2 (29.1)
GOLD (Ⅰ/Ⅱ/Ⅲ/Ⅳ), n	13/13/17/4	7/6/6/0	1/2/3/0	2/4/3/2	3/1/5/2
mMRC	2.0 [1.0, 2.0]	2.0 [1.0, 2.0]	1.0 [1.0, 2.0]	2.0 [1.5, 3.0]	2.0 [1.0, 3.0]
CAT, points	14.2 (8.4)	9.9 (6.0)	20.5 (10.2)	12.8 (6.2)	19.4 (8.9)
6MWD, m	343.6 (145.8)	448.7 (88.8)	325.2 (172.3)	271.1 (108.3)	254.1 (144.7)
QF, kg	36.5 (14.4)	46.5 (13.4)	26.5 (9.6)	33.7 (10.0)	27.5 (11.3)
MIP, cmH_2O	66.2 (29.4)	90.1 (22.2)	81.6 (14.0)	43.2 (14.0)	39.6 (14.8)
Daily steps, step/day	3912 (3331)	5194 (3364)	5267 (5020)	2600 (2051)	2235 (2299)
MNA-SF, points	10.8 (2.5)	12.2 (1.4)	9.3 (1.2)	11.8 (1.3)	8.1 (3.2)
KCL, points	0.2 (0.4)	0.1 (0.2)	0.2 (0.4)	0.2 (0.4)	0.4 (0.5)
J-CHS score, points	1.1 (1.1)	0.6 (0.8)	1.5 (1.6)	1.1 (0.8)	1.8 (1.1)

Data expressed as absolute frequency, mean (SD) or median [IQR 25–75%]. MW, Muscle Wasting; RW, Respiratory Weakness; BMI, body mass index, FFMI, fat-free mass index; VC, vital capacity; FVC, forced vital capacity; FEV_1, forced expiratory volume in the first second; GOLD, Global Initiative for Chronic Lung Disease; mMRC, modified Medical Research Council; 6MWD, six-minute walk distance; CAT, COPD Assessment Test; HG, hand grip; QF, quadriceps femoris muscle force; MIP, maximum inspiratory pressure; MNA-SF, Mini Nutritional Assessment-short form; KCL, Kihon checklist ; J-CHS, Japanese version of Cardiovascular Health Study criteria.
